# Analysis of Microbial Communities and Microbial Preservation of the Qilin Screen Wall and Text Brick Wall in the Jinshanling Great Wall

**DOI:** 10.3390/microorganisms14051056

**Published:** 2026-05-08

**Authors:** Zhiqian Guan, Yu Wang, Yibo Geng, Yuanyuan Wang, Lilong Hou, Xingling Tian, Jiao Pan

**Affiliations:** 1 Key Laboratory of Archaeomaterials and Conservation, Ministry of Education, University of Science and Technology Beijing, Beijing 100083, China; 18111541120@163.com (Z.G.); d202310766@xs.ustb.edu.cn (Y.W.); m202511462@xs.ustb.edu.cn (Y.G.); wyy041220@gmail.com (Y.W.); 2 Institute for Cultural Heritage and History of Science & Technology, University of Science and Technology Beijing, Beijing 100083, China; 3 College of Life Sciences, Nankai University, Tianjin 300071, China; 2120221471@nankai.edu.cn; 4 China Academy of Cultural Heritage, Beijing 100029, China; 13520238749@163.com

**Keywords:** masonry ancient architecture, biodeterioration, microbial community, antimicrobials, cultural relic conservation

## Abstract

The Jinshanling Great Wall is an important part of the Ming Great Wall, the most important material cultural heritage of China, and is currently facing a significant threat of microbial degradation due to the widespread biological weathering of open-air masonry buildings. This study focuses on the Qilin Screen Wall and Text Brick Wall of the Jinshanling Great Wall, utilizing scanning electron microscopy (SEM) and metabarcoding analyses to reveal the diverse microbial communities coexisting on the masonry surfaces, including various lichens, molds, and bacteria. Twelve fungal strains were successfully isolated. The antimicrobial experiment results indicated that 0.6% isothiazolinone-based antimicrobial BC01, 50 mg/mL carvacrol and 50 mg/mL thymol exhibited a certain degree of antimicrobial activity against these strains. Overall, this study has laid a solid foundation for microbial control of the masonry Great Wall through in-depth analysis of microbial community structure and screening of highly effective antimicrobials.

## 1. Introduction

**Figure 1:**
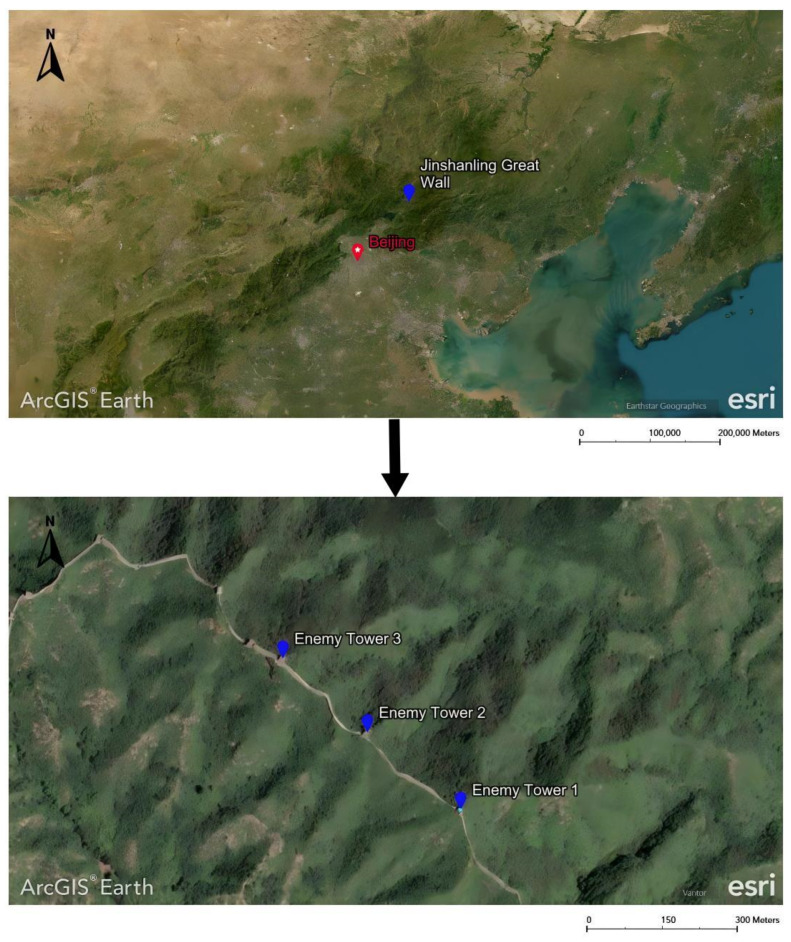
Geographical location of the Jinshanling Great Wall.

The Ming Great Wall is a prominent representative of the remains of the Great Wall in China [1]. It was mainly constructed of brick and stone, located in northern China, and is in relatively good condition [2]. In the middle of the Ming Dynasty, the Great Wall served as the northern border, and the government established “Nine Military Districts of the Frontier” (九边重镇 *Jiubian Zhongzhen*) to divide jurisdiction. The Jinshanling Great Wall belongs to one of these districts, specifically Jizhou, located in Luanping County, Chengde City, Hebei Province. It is about 15 km long and has 67 enemy towers. It is the most complex and densely packed section of the Great Wall, with profound archeological value, and is of great significance for studying the history of the Ming Dynasty in China and the life of Qi Jiguang. The Jinshanling Great Wall has four wonders, namely the Qilin Screen Wall, the Text Brick Wall, the Barrier Wall, and the Horse Barrier Wall. Among them, the Qilin Screen Wall, located on enemy tower 2, is a stone structure adorned with the qilin motif crafted from black bricks. It remains the only intact qilin painting preserved in the Ming Great Wall architectural complex to date. The Text Brick Wall lies between enemy towers 1 and 2, featuring multiple text bricks that display information about different construction units in regular script and running script (Figure 1). Due to its long history and exposure to outdoor conditions, this section of the Great Wall faces severe weathering issues, among which biological degradation (particularly microbial growth and metabolic activities) has become a significant factor that cannot be overlooked.

In recent years, the adoption of high-throughput sequencing for studying microbial communities on cultural heritage surfaces has gained increasing popularity [3,4]. It is widely accepted that bacteria, archaea, fungi, algae, and lichens can exist on the surfaces of lithic relics under various environmental conditions and in various geographical regions [5,6]. High-throughput sequencing enables comprehensive analysis of the composition and structure of complex microbial communities on relic surfaces, whereas traditional culture methods can only isolate microbial taxa that constitute a negligible proportion in natural environments. Through metabarcoding analysis, researchers can rapidly and accurately reveal the composition and relative abundance of microbial communities, providing a scientific basis for cultural heritage conservation efforts [7].

Although ancient architecture, along with grotto temples and stone carvings, belongs to the category of cultural relics and differs in craftsmanship, from a biological perspective, the materials of masonry ancient architecture are similar to those of the latter two. The colonization of organisms on rocks can be defined as bioreceptivity, which is primarily related to parameters such as the mineral type, chemical composition, roughness, porosity, and permeability of stone cultural relics [8,9]. The biological receptivity of aged or deteriorated stone materials is generally higher than that of fresh stone materials [10], making them more susceptible to microbial colonization and damage. Biodegradation is a slow process initiated by pioneer microorganisms such as cyanobacteria, algae, and lichens, which modify stone properties by forming biofilms and subsequently induce the colonization of heterotrophic bacteria and fungi [11]. Biofilms colonized on the surface of stone may have multiple colors, which seriously affects the esthetic value of stone artifacts [12]. In addition, the presence of epilithic biofilms can also bring about physical and chemical changes, corrosion by organic acids, and secondary mineralization [11]. The impact of the above organisms on stone is collectively referred to as “biodeterioration”, but “bioprotection” also occurs. This is reflected in the positive role that biofilms can play in regulating water and temperature changes to reduce freeze–thaw damage to stone [13], or directly protect stone from wind and rain erosion [14]. In addition, fungi or lichens can induce biomineralization (such as calcium carbonate and calcium oxalate) to form protective coatings [15]. The biocrust, composed of cyanobacteria, moss, lichen, other microorganisms and tightly bound soil particles, is widely distributed along the rammed-earth Great Wall in Northwest China [16]. Relevant studies have confirmed that biocrusts provide long-term and multifaceted erosion protection for the Great Wall by enhancing mechanical stability and reducing the erosiveness of the rammed earth [17]. A criterion has been proposed to determine the ambivalent role of biofilms on stone artifacts, based on an assessment of the dominance between natural weathering and biodegradation [18]. Currently, the debate on bioprotection versus biodeterioration remains intense. To control biodeterioration, antimicrobials have been widely applied in the protection of stone cultural heritage, but their use for mitigating biodeterioration is controversial and should be employed with caution [19,20,21].

In this study, we investigated the Qilin Screen Wall and two sections of the Text Brick Wall between enemy towers 1 and 2 in Jinshanling, and found that due to natural and human factors, the patterns and texts of cultural relics were severely damaged. The core of the wall is severely weathered and there are safety hazards in the wall structure. The phenomenon of microbial colonization is particularly prominent. In order to explore the harm of microorganisms to walls and evaluate the impact of removing these microorganisms, it is necessary to first study the microbial communities on the Qilin Screen Wall and Text Brick Wall. This work utilized microbiology and molecular biology techniques to reveal the composition and structure of microbial communities on the surface of masonry cultural relics. This research helps provide a basis for the prevention and protection of microorganisms and the reinforcement and repair of walls.

## 2. Materials and Methods

### 2.1. Field Sites and Sample Collections

The field investigation was conducted in September 2023 on the Text Brick Wall between enemy towers 1 and 2 of the Jinshanling Great Wall and the Qilin Screen Wall on enemy tower 2. The Great Wall in this section runs roughly from northwest to southeast. The Qilin Screen Wall, positioned on the southwest side, measures 1.84 m in length and 1.1 m in height, consisting of three components: the top section, the body section, and the base section. The Qilin pattern consists of 15 identical square bricks arranged in a 5-by-3 grid. It is shielded from intense sunlight for most of the time. The environmental temperature at the sampling site was 15 °C, with a relative humidity of 68%. We selected microorganisms at different locations on the Text Brick Wall and Qilin Screen Wall, with colors ranging from gray, green, yellow, and brown, and shapes in clusters and shells. They were scraped off with a sterile surgical knife for high-throughput sequencing. Carbon conductive adhesive was used to adhere these microorganisms for subsequent scanning electron microscopy observation. In addition, sterile cotton swabs were used to dip these microorganisms and apply them onto PDA culture medium. A total of 7 samples were collected in this survey, with 5 from the Qilin Screen Wall (designated as C1 to C5) and 2 from the Text Brick wall (designated as C6 and C7). The field sampling are shown in Table 1.

### 2.2. Microscopic Observation

Sample morphology was observed using a Quanta 200 scanning electron microscope (FEI, Hillsboro, OR, USA). Samples were fixed onto conductive carbon adhesive, dried in a desiccator, and mounted onto SEM stubs. Gold sputtering was conducted at 24 mA for 300 s [22]. Microscopic imaging was performed at an accelerating voltage (EHT) of 20.0 kV, with a working distance (WD) of 10.3–11.2 mm and magnification ranging from 1000× to 10,000×. A total of 7 samples were analyzed, including 5 samples from the Qilin Shadow Wall and 2 samples from the Text Brick Wall.

### 2.3. Amplicon Sequencing and Metagenomic Sequencing Analysis

#### 2.3.1. Total DNA Extraction and Library Preparation

Total microbial genomic DNA was extracted from samples using the DNeasy PowerSoil Kit (QIAGEN, Netherlands). The extracted DNA was sent to Novogene Technology Co., Ltd. (Beijing, China) for high-throughput sequencing. The 16S rRNA V4 region was amplified using primers 515F (5′-GTGCCAGCMGCCGCGGTAA-3′) and 806R (5′-GGACTACHVGGGTWTCTAAT-3′) for prokaryotic community profiling [23]. The 18S rRNA V4 region was amplified using primers 528F (5′-GCGGTAATTCCAGCTCCAA-3′) and 706R (5′-AATCCRAGAATTTCACCTCT-3′) for eukaryotic community analysis [24]. Sequencing libraries were prepared using the TruSeq^®^ DNA PCR-Free Sample Preparation Kit (Illumina, USA). Library concentration and quality were validated using a Qubit fluorometer, real-time PCR, and a Bioanalyzer. Equimolar libraries were pooled and sequenced on an Illumina platform. Raw sequencing data were deposited in the NCBI Sequence Read Archive (SRA) under BioProject accession number PRJNA1366773.

#### 2.3.2. ASVs Denoise, Species Annotation and Visualization

Effective tags were processed using the QIIME2 platform (version 202202). Denoising was performed using the DADA2 or deblur module to generate amplicon sequence variants (ASVs). Taxonomic annotation was carried out in QIIME2 against the Silva database for 16S/18S rRNA genes. Venn diagrams were constructed in R using the VennDiagram package. Relative abundance histograms of the top 10 taxa at each taxonomic level were generated in Perl using the SVG function. Phylogenetic trees were constructed based on the 100 most abundant genera after sequence alignment and visualized in Perl using the SVG function [25].

#### 2.3.3. Alpha Diversity and Beta Diversity Analysis

Alpha diversity was evaluated in QIIME2 using seven indices: Observed_features, Chao1, Shannon, Simpson, Dominance, Goods_coverage, and Pielou_e to assess community richness, diversity, and evenness. Beta diversity was calculated based on weighted UniFrac distances in QIIME2 to compare community dissimilarity among samples. A species abundance table was generated by merging feature sequences at the same taxonomic level. Weighted UniFrac distance matrices were constructed using phylogenetic relationships and feature abundance [26,27,28]. Principal co-ordinates analysis (PCoA) and non-metric multi-dimensional scaling (NMDS) were applied to visualize sample differentiation. PCoA and NMDS plots were constructed in R (version 4.0.3) using the ade4 and ggplot2 packages.

#### 2.3.4. Association Analysis

To reveal species co-occurrence patterns and environmental impacts on community structure, two-dimensional and three-dimensional association networks were constructed. Correlations were further examined using Spearman’s correlation analysis. After calculating the Spearman correlation index for all samples and obtaining the species correlation coefficient matrix, the filtering conditions were set as follows: (1) remove connections with correlation coefficients < 0.6; (2) filter out node self-connections; and (3) remove connections with node abundance less than 0.005%, and then obtain the network graph. Network analysis was performed in R using the igraph, psych, tidyr, and dplyr packages, and visualized with Graphviz software (version 2.38.0).

### 2.4. Isolation, Purification and Identification of Cultivable Fungi

Field-sampled fungi were cultured on PDA medium (Qingdao Hope Bio-Technology Co., Ltd., Qingdao, China) at 28 ± 1 °C until visible colonies emerged. Colony morphology and color were recorded, and distinct colonies were transferred to fresh PDA plates using an inoculation loop. Pure strains were obtained after incubation at 28 °C for 3 days.

The hyphae of pure strains were collected, and a small amount of hyphae was added to lysis buffer for lysis. The genomic DNA was extracted using a phenol/chloroform method. DNA concentration and purity were measured using a DS-11FX+ ultra-micro spectrophotometer/fluorometer (Beijing Bio-Sun Technology Co., Ltd., Beijing, China). The ITS region was amplified with primers ITS1 (5′-TCCGTAGGTGAACCTGCGG-3′) and ITS4 (5′-CCTCCGCTTATTGATATGC-3′) [29]. PCR was performed in a 25 μL mixture containing 12.5 μL 2 × GS Taq PCR Mix (Genesand Biotech, Beijing, China), 2 μL forward primer, 2 μL reverse primer, and 2 μL template DNA. The thermal program included an initial denaturation at 95 °C for 3 min, followed by 35 cycles of 94 °C for 25 s, 57 °C for 25 s, 72 °C for 10 s, and a final extension at 72 °C for 5 min. PCR products were verified by 2% agarose gel electrophoresis and sequenced by GENEWIZ (Beijing, China). Sequences were compared using the NCBI BLAST tool (version Blast+ 2.16.0). All fungal sequences were deposited in GenBank under accession numbers PX600449–PX600460.

### 2.5. Antimicrobial Experiment

#### 2.5.1. Chemical Antimicrobials

Antimicrobial activities of isothiazolinone derivatives and traditional Chinese medicine antimicrobials were evaluated using the filter paper disk method against purified fungal isolates. Fungal spores were evenly spread onto PDA medium. Three or four sterile filter papers were placed on each plate with consistent spacing. Ultrapure water or dimethyl sulfoxide (DMSO) was applied to one filter paper as a negative control, and 5 μL of each antimicrobial solution was added to the remaining disks. Plates were incubated at 28 ± 1 °C for 48 h, and inhibition zones were measured.

#### 2.5.2. Nano-Metal Oxides

A suspension quantitative bactericidal assay was used to assess the antimicrobial effects of two nano-metal oxides. Antimicrobial materials were pre-irradiated under ultraviolet light for 30 min. Sterile water and test materials were mixed in sterilized centrifuge tubes, followed by the addition of fungal suspension (1 × 10^5^ cells/mL) with thorough mixing. After incubation, 50 μL of the mixture was transferred onto solid medium and spread evenly. Plates were sealed and incubated at 28 °C for 48 h, and colony survival rates were recorded. The names and concentrations of antimicrobials are listed in Table 2.

## 3. Results

### 3.1. SEM Results of Samples from the Surface of the Qilin Screen Wall and Text Brick Wall

**Figure 2:**
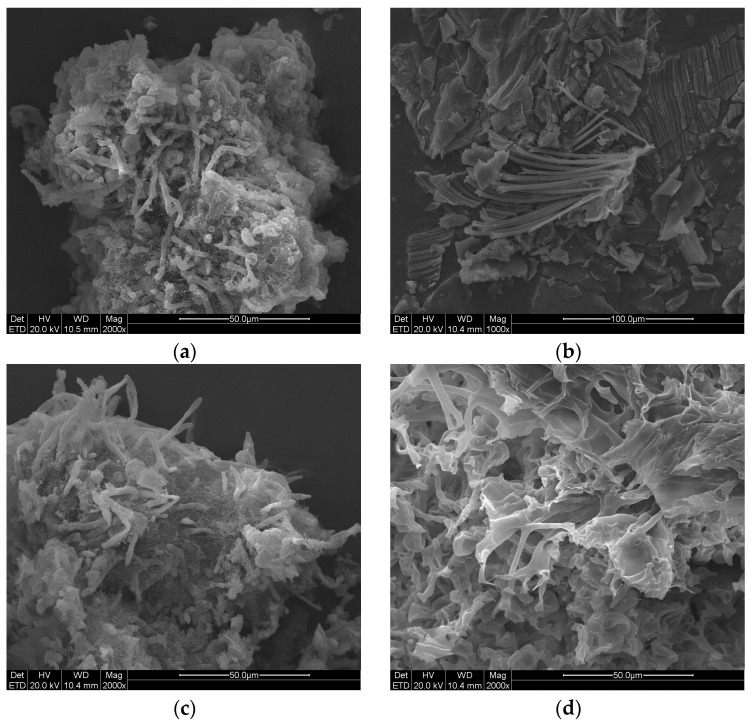

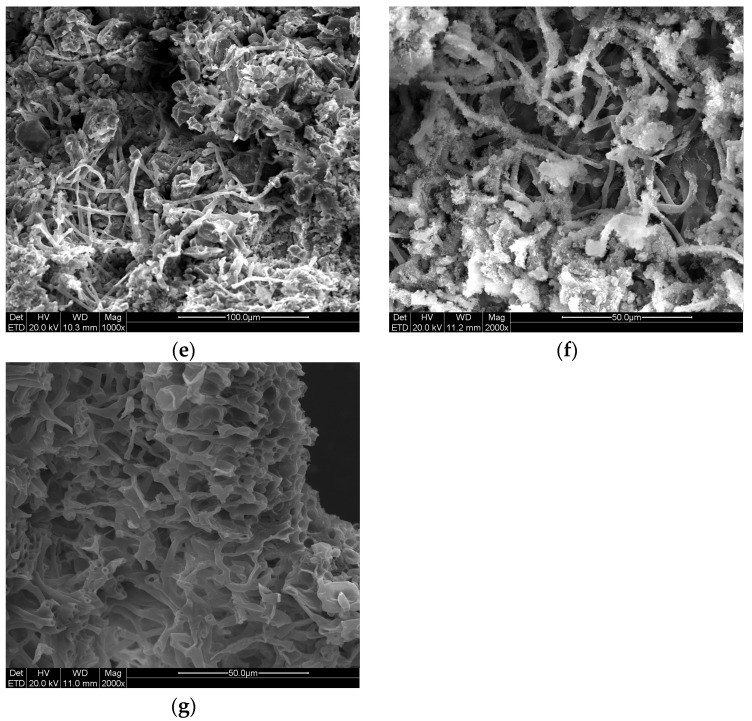
SEM images of biological samples collected from the Jinshanling Great Wall: (**a**–**e**) represent samples C1 to C5 from the Qilin Screen Wall, respectively; (**f**) and (**g**) represent samples C6 and C7 from the Text Brick Wall, respectively.

Some slender filamentous structures can be seen in samples C1 to C7 under SEM, as shown in Figure 2. The diameter of these filamentous structures is only a few microns, which conforms to the scale of fungal hyphae. In sample C2 (Figure 2b), these hyphae appear to be originally arranged in a relatively tight and regular manner. Around the larger diameter hyphae, numerous extremely fine filaments with a diameter of approximately 0.2 μm can be observed. These are presumed to be Actinomycete mycelia, primarily found in samples C1 and C3 (Figure 2a,c). Samples C4 and C7 (Figure 2d,g) show highly porous, three-dimensionally interconnected network structures, displaying an overall microstructure resembling a “sponge-like” morphology. In sample C5 (Figure 2e), numerous slender, branched hyphal structures are observed, with some showing umbrella-shaped apical bulging and others merging into intertwined clusters. The darker areas contain irregular blocky and clastic structures, which are presumed to be substrates in the microbial growth environment. In sample C6 (Figure 2f), the surfaces of many hyphae are not smooth but covered with a blurred, flocculent substance that appears relatively bright. No spores were clearly observed in these samples, which differs from some previous cultural relic detection analyses conducted by our research team [22,30]. This discrepancy may be attributed to the nutrient-deficient surface of the bricks and stones. The visible microbial colonies on the relics are the result of complex synergistic interactions among multiple microbial species, rather than the growth of a single organism.

### 3.2. Cultivable Fungi Isolation, Cultivation, and Identification

**Figure 3:**
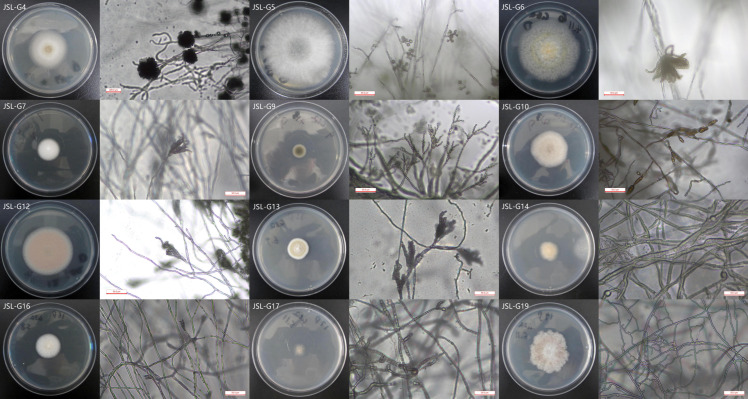
Plate photos of isolated and purified fungi (the inner diameter of the medium is 8.7 cm).

In September 2023, we collected a total of seven microbial samples on the surface of the Text Brick Wall between enemy towers 1 and 3, and the Qilin Screen Wall on enemy tower 2 of the Jinshanling Great Wall. Following separation and purification, 12 cultivable fungi were identified and classified, as shown in Table 3. The plate photographs and photomicrographs of the fungi isolated are presented in Figure 3.

### 3.3. Microbial Community Analysis

#### 3.3.1. Species Visualization

**Figure 4:**
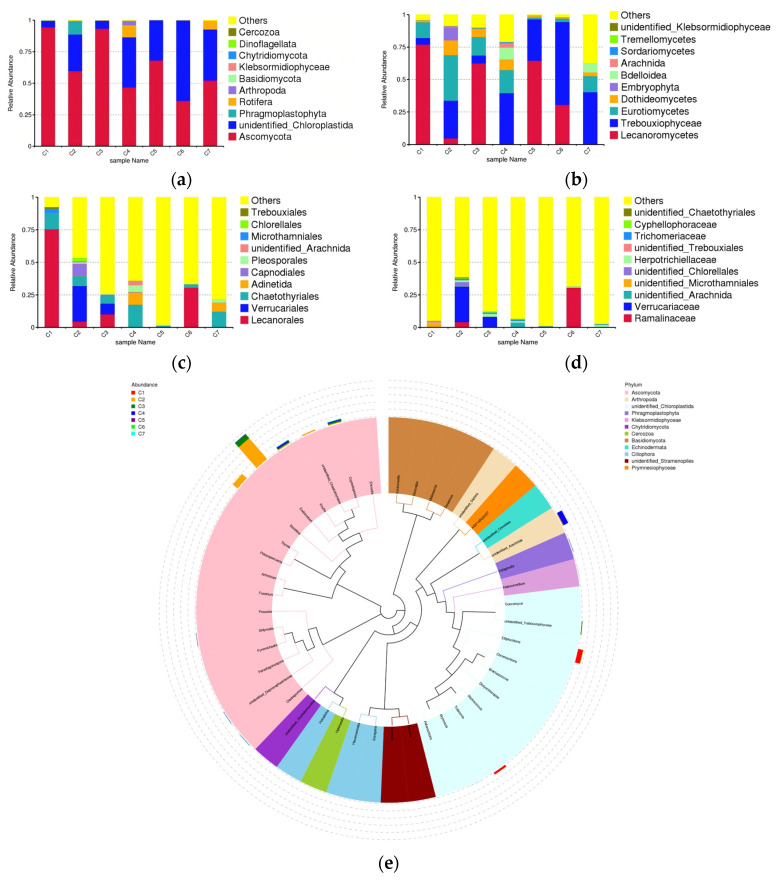
Relative abundance of eukaryotic microorganisms in different samples: (**a**): the phylum-level diversity results; (**b**): the class-level diversity results; (**c**): the order-level diversity results; (**d**): the family-level diversity results; (**e**): the phylogenetic tree at the genus level. Others: The corresponding levels exclude all species except those with the top 10 abundance rankings, including species ranked 11th and below, as well as species without annotated information at that level.

The sequencing results show that at the phylum level (Figure 4a), Ascomycota and unidentified_Chloroplastida are the dominant phyla across all samples. In samples C1 and C3, unidentified_Chloroplastida is significantly less abundant than Ascomycota. In sample C6, the proportion of unidentified_Chloroplastida (63.69%) exceeds that of Ascomycota (36.21%). Phragmosplastophyta is only detected in sample C2, while Athropoda is only detected in sample C4. Samples C4 and C7 also contain a sufficient number of representatives of Rotifera. At the class level (Figure 4b), Lecanoromycetes is the dominant across samples C1, C3 and C5, whereas in samples C4, C6 and C7, it is Trebouxiophyceae, and in sample C2, it is Eurotiomycetes. In addition, Dothideomycetes shows sufficient representation in samples C2, C3, C4 and C7. Embryophyta is only detected in sample C2, while Arachnida is only detected in sample C4. Samples C4 and C7 also contain a sufficient number of representatives of Bdelloidea. At the order level (Figure 4c), Lecanorales is the dominant across samples C1, C3 and C6, whereas in samples C4 and C7, it is Chaetothyriales. Chaetothyriales is also abundantly detected in samples C1, C2, C3 and C6. For sample C2, the dominant order is Verrucariales, which accounts for the second-largest proportion in sample C3. In addition, Capnodiales is only detected in sample C2, while unidentified_Arachnida is only detected in sample C4. Samples C4 and C7 also contain a sufficient number of representatives of Adinetida and Pleosporales. At the family level (Figure 4d), Verrucariaceae is the dominant in samples C2 and C3, whereas in sample C6, it is Ramalinaceae, which accounts for the second-largest proportion in sample C2. For sample C1, the dominant taxon is unidentified_Microthamniales, while in sample C4, it is unidentified_Arachnida. The phylogenetic tree (Figure 4e) reveals a higher abundance of *Elliptochloris* and *Trebouxia* (unidentified_Chloroplastida) in sample C1. The abundant taxa in sample C2 includes *Endocarpon* and *Bacidina* belonging to Ascomycota. Sample C3 also contains a certain abundance of *Endocarpon*. Another genus detected in this sample, *Cyphellophora*, belongs to the phylum Ascomycota as well. The abundant taxa in sample C4 include unidentified_Arachnida belonging to Arthropoda, as well as *Knufia* belonging to Ascomycota. The percentage of the top ten eukaryotic taxa by relative abundance at each taxonomic level is presented in Supplementary Table S1.

**Figure 5:**
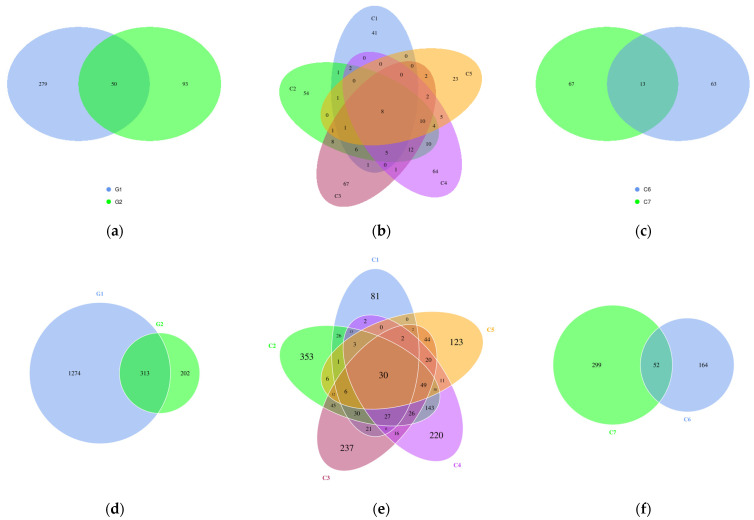
Analysis of the number of common and unique eukaryotic and prokaryotic microbial characteristic sequences: (**a**): The Venn diagram of two groups of samples (eukaryotic); G1 represents the Qilin Screen Wall, G2 represents the Text Brick Wall. (**b**): The Venn diagram of the five samples of G1 (eukaryotic). (**c**): The Venn diagram of the two samples of G2 (eukaryotic). (**d**): The Venn diagram of two groups of samples (prokaryotic); G1 represents the Qilin Screen Wall, G2 represents the Text Brick Wall. (**e**): The Venn diagram of the five samples of G1 (prokaryotic). (**f**): The Venn diagram of the two samples of G2 (prokaryotic).

Venn analysis of common and unique characteristic sequences in the collected samples from the two locations was performed, provided that the number of samples (groups) was less than or equal to 5. As shown in Figure 5a, the Qilin Screen Wall contains more unique characteristic sequences than the Text Brick Wall, whereas the common characteristic sequences are fewer than their unique characteristic sequences. As shown in Figure 5b, the five samples of the Qilin Screen Wall each contain a large number of unique characteristic sequences, and the proportion of common characteristic sequences is extremely low. Figure 5c also reflects this characteristic in the Text Brick Wall.

**Figure 6:**
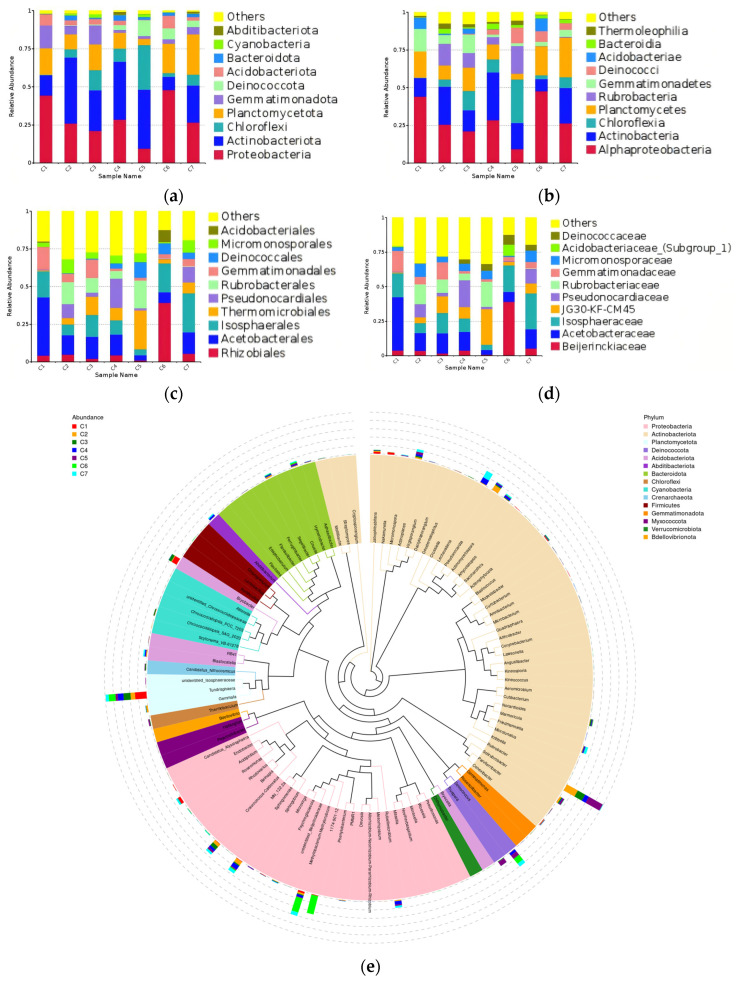
Relative abundance of prokaryotic microorganisms in different samples: (**a**): the phylum-level diversity results; (**b**): the class-level diversity results; (**c**): the order-level diversity results; (**d**): the family-level diversity results; (**e**): the phylogenetic tree at the genus level. Others: The corresponding levels exclude all species except those with the top 10 abundance rankings, including species ranked 11th and below, as well as species without annotated information at that level.

The prokaryotic diversity results obtained from the above-mentioned seven samples were also analyzed. The results are shown in Figure 6. At the phylum level (Figure 6a), Proteobacteria (currently Pseudomonadota) and Actinobacteriota are the dominant groups. Planctomycetota and Gemmatimonadota are detected abundantly across all samples. Except for sample C1, Chloroflexi (currently Chloroflexota) shows high relative abundance in all samples. In addition, Deinococcota and Acidobacteriota are also detected in a sufficient number of representatives across several samples. At the class level (Figure 6b), the proportions of the top ten taxa by relative abundance nearly correspond precisely to those of their higher-level taxa at the phylum level. The only difference lies in the detection of Rubrobacteria in the C2, C3, C4, and C5 samples. This class and Actinobacteria (currently Actinomycetes) both belong to Actinobacteriota. At the order level (Figure 6c), for samples C1, C5, C6, and C7, the dominant ones are Acetobacterales, Thermomicrobiales, Rhizobiales and Isosphaerales, respectively. For the remaining four samples, none of the taxa showed a particularly dominant proportion. The taxa ranked 5th to 9th are detected in large numbers across several samples. Acidobacteriales is exclusively and distinctly detected in sample C6. Overall, sample C1 has the simplest composition among all samples. At the family level (Figure 6d), for samples C1, C5, C6, and C7, the dominant ones are Acetobacteraceae, JG30-KF-CM45, Beijerinckiaceae and Isosphaeraceae, respectively. The overall composition of all samples closely matches the situation at the order level. The phylogenetic tree (Figure 6e) reveals a higher abundance of *Rubrobacter*, *Methylobacterium*-*Methylorubrum*, *Tundrisphaera* and *Deinococcus* in several samples. Apart from these four taxa, the abundant taxon in sample C1 is *Bryobacter*, which belongs to Acidobacteriota. The abundant taxa in sample C2 are *Pseudonocardia* (Actinobacteriota) and *Sphingomonas* (Proteobacteria). Sample C4 and C7 also contain a certain abundance of *Sphingomonas*, and in these two samples, *Lechevalieria* (Actinobacteriota) shows high relative abundance. The abundant taxon in sample C6 is 1174-901-12 (Rhizobiales). The percentage of the top ten prokaryotic taxa by relative abundance at each taxonomic level is presented in Supplementary Table S2.

The characteristics presented in the results of Venn analysis of common and unique characteristic sequences are similar to those of eukaryotic microorganisms, as shown in Figure 5, and the only difference is that the unique characteristic sequences of the Text Brick Wall are fewer than the common characteristic sequences (Figure 5d), while the opposite is true in eukaryotic microorganisms (Figure 5a).

#### 3.3.2. Alpha and Beta Diversity

Alpha diversity is used to analyze the diversity of microbial communities within a sample [31]. Diversity analysis of a single sample can reflect the abundance and diversity of microbial communities within the sample, including using a series of statistical analysis indices and species diversity curves to evaluate the differences in species abundance and diversity of microbial communities in each sample. The statistical results of the alpha diversity index are presented in Table 4 and Table 5. The species richness (chao1) of samples C2, C3 and C4 is significantly higher than that of the other four samples. Meanwhile, based on the dominance index, pielou_e index, and Shannon index, the species evenness and overall diversity of these three samples are also relatively high. For sample C5, its eukaryotic species richness is the lowest, and the eukaryotic species evenness is low. However, its prokaryotic species evenness and overall diversity are comparable to those of sample C4. Sample C7 shows moderate levels of species richness, evenness, and overall diversity among all samples. For samples C1 and C6, they rank among the lowest in both species evenness and overall diversity. The Simpson index of 18S reflects the evenness of species among samples, and its relative value corresponds to that indicated by the dominance index. In contrast, the Simpson index of 16S for each sample is very close. The rarefaction curves established based on these diversity indices are shown in supplementary Figures S1 and S2, which indicate that sufficient sequencing depth had been achieved.

**Figure 7:**
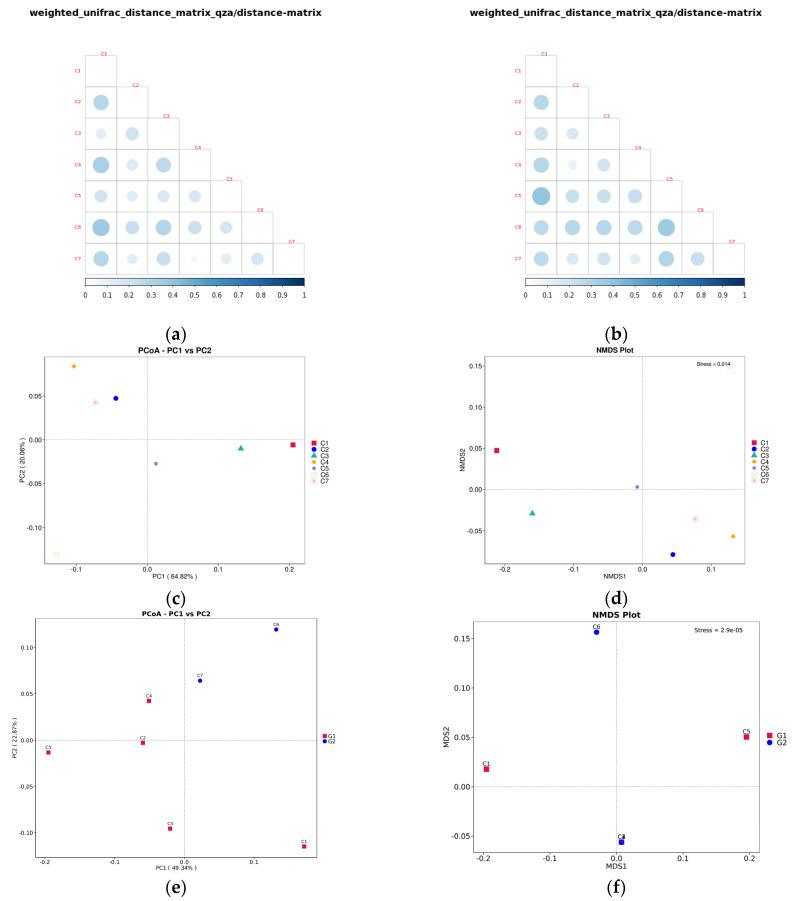
Analysis of beta diversity of eukaryotic microorganism: (**a**): Eukaryotic beta diversity index. (**b**): Prokaryotic beta diversity index. (**c**): 2D PCoA based on weighted_unifrac distance (eukaryotic); the horizontal axis represents one principal component, the vertical axis represents another principal component, and the percentage indicates the contribution of the principal component to the sample variance. Each point in the plot represents a sample. (**d**): NMDS based on weighted_unifrac distance (eukaryotic); each dot in the figure represents a sample, with the distance between dots indicating the degree of variation. Samples within the same group are colored uniformly. When the stress value is less than 0.2, it indicates that NMDS accurately reflects the differences between samples. (**e**): 2D PCoA based on weighted_unifrac distance (prokaryotic). (**f**): NMDS based on weighted_unifrac distance (prokaryotic).

The beta diversity analysis is shown in Figure 7, including the beta diversity index, PCoA and NMDS. For the 18S analysis results, as shown in Figure 7a, the diversity of sample C4 is remarkably similar to that of C7. Sample C1 shows significant differences compared to samples C2, C4, C6, and C7. Sample C3 shows differences compared to samples C4 and C6. For the 16S analysis results, as shown in Figure 7b, sample C2 shows the closest diversity to sample C4, followed by sample C7. Sample C5 shows significant differences compared to samples C1 and C6. These characteristics are also evident in both PCoA and NMDS analyses. For the results of 18S, the points representing samples C4 and C7 are very close to each other on the coordinate graph, while the point representing sample C1 is very far from those of samples C2, C4, C6 and C7. Similarly, the point of sample C3 is also far from those of samples C4 and C6 (Figure 7c,d). For the results of 16S, the points of samples C2, C4, and C7 are closely spaced together. And the points in samples C1, C5, and C6 are separated by considerable distances from each other (Figure 7e). In the NMDS analysis, the five points except C1, C5, and C6 nearly coincided (Figure 7f).

### 3.4. Association

**Figure 8:**
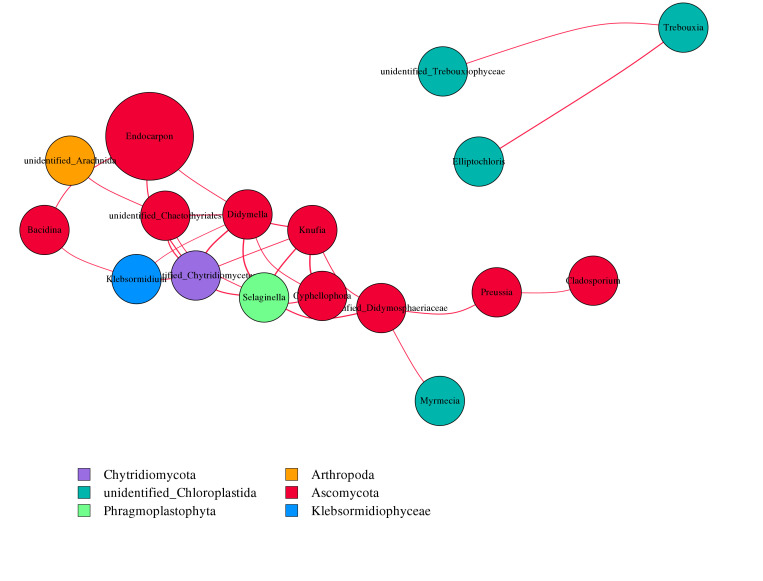
The network of eukaryotic microbial association analysis (different nodes represent different genera, and node size represents the average relative abundance of that genus; nodes in the same phylum have the same color (as shown in the legend)). The thickness of the connections between nodes is positively correlated with the absolute value of the correlation coefficient between species interactions, and the color of the connections corresponds positively or negatively to the correlation (red positive correlation, blue negative correlation).

As shown in Figure 8, both *Didymella* (Ascomycota) and *Selaginella* (Phragmoplastophyta) are correlated with the other six genera, respectively, followed by *Knufia* (Ascomycota) and unidentified_Chaetothyriales (Ascomycota), which are associated with five genera, respectively. The three genera in the upper right corner all belong to unidentified_Chloroplastida, which naturally gather together. The association analysis of prokaryotes is presented in supplementary Figure S3; the vast majority of genera are positively correlated with each other, while the only genera that show a negative correlation with all others are *Pseudomonas* (Proteobacteria, currently Pseudomonadota) as well as *Curtobacterium* (Actinobacteriota, currently Actinomycetota). *Bryobacter* (Acidobacteriota), *Deinococcus* (Deinococcota), and *Methylobacterium*-*Methylorubrum* (Proteobacteria) shows positive correlations with some genera and negative correlations with others.

### 3.5. Antimicrobial Experiment

#### 3.5.1. Chemical Antimicrobials

**Figure 9:**
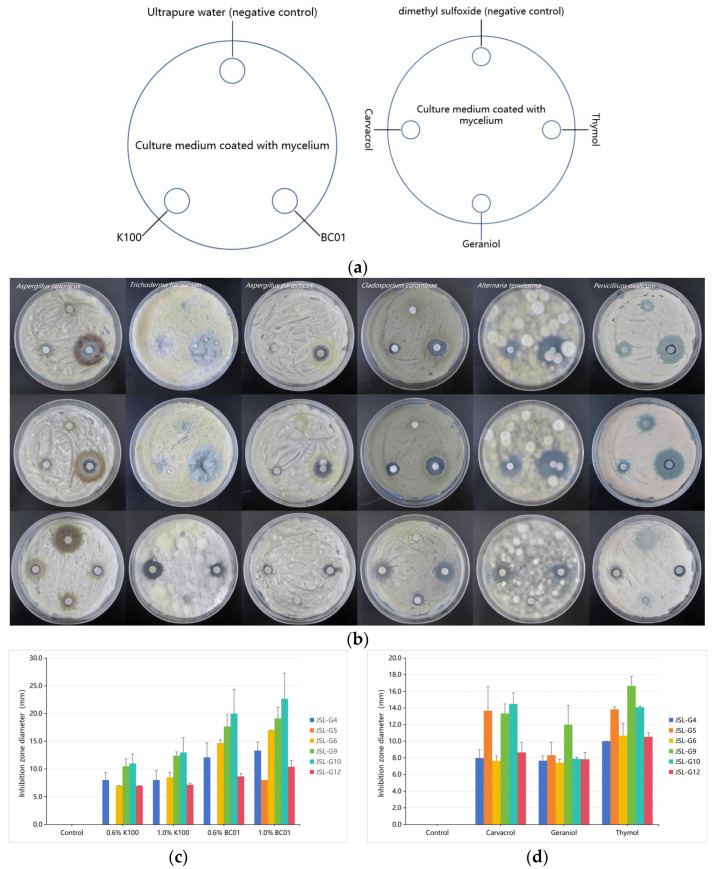
Inhibitory effect of different antimicrobials on different strains: (**a**): A schematic diagram of antimicrobial experiment results. (**b**): The actual antimicrobial efficacy, and the first line is 0.6% K100 and BC01, the second line is 1.0% K100 and BC01, and the third line are three traditional Chinese medicine compound. (**c**): The size of inhibition zone produced by K100 and BC01; (**d**): the size of inhibition zone produced by Carvacrol, Geraniol and Thymol.

The selection of antimicrobial concentration (Table 2) is based on previous multiple studies involving the use of K100, BC01, and essential oil components [30,32]. As shown in Figure 9, all fungal plates were the results after two days of cultivation. Figure 9c shows that under the same concentration, the inhibitory effect of BC01 on fungi is superior to that of K100. The two antimicrobials show weak inhibitory effects on *Penicillium oxalicum*, while have almost no effect on *Trichoderma harzianum*; only relatively slow growth of fungi was observed in the filter paper area. Figure 9d shows that under the same concentration, among the six fungi, only *Cladosporium colombiae* can be effectively inhibited by geraniol, while *Aspergillus parasiticus* can only be effectively inhibited by thymol. Overall, among the three antimicrobials, thymol has the best inhibitory effect on these strains, while carvacrol is slightly inferior.

#### 3.5.2. Nano Mental Oxides

**Figure 10:**
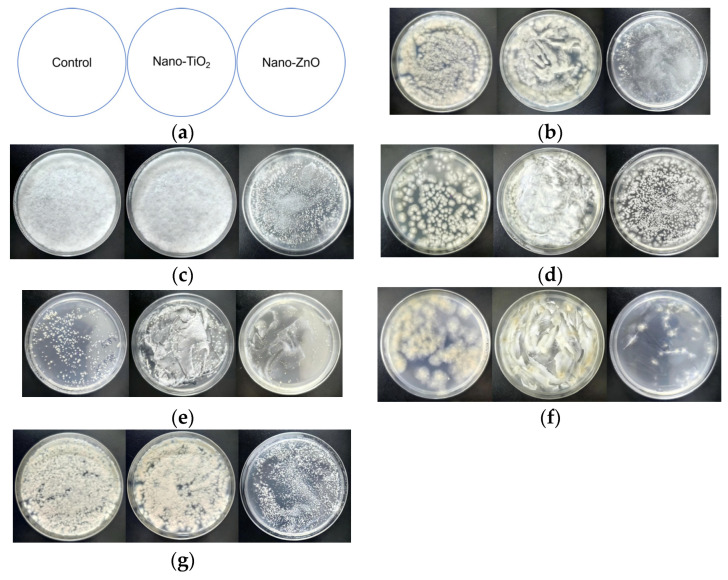
Inhibitory effect of nanomaterials on fungal spore germination. (**a**): A schematic diagram of antimicrobial experiment results; (**b**): *Aspergillus japonicus*; (**c**): *Trichoderma harzianum*; (**d**): *Aspergillus parasiticus*; (**e**): *Cladosporium colombiae*; (**f**): *Alternaria tenuissima*; (**g**): *Penicillium oxalicum*.

As shown in Figure 10, nano-ZnO shows significant inhibitory effects on spore germination of the other five fungi, except for *A. parasiticus*. Although the spores of *A*. *parasiticus* germinated more frequently, the colonies formed were still less colored than those on the control plate. Nano-TiO_2_ shows no inhibitory effect.

## 4. Discussion

Bioinformatics analysis was performed on microbial samples from the Qilin Screen Wall and Text Brick Wall of the Jinshanling Great Wall. The species diversity and species association of the microbial community were analyzed. Subsequently, we conducted antimicrobial experiments. This study provides basic data for the prevention and control of microbial degradation in the Qilin Screen Wall and Text Brick Wall of the Jinshanling Great Wall, which is of great significance for the biodeterioration control of outdoor immovable masonry ancient architecture.

### 4.1. Analysis of the Composition of Microbial Community Structure in the Qilin Screen Wall and Text Brick Wall of the Jinshanling Great Wall

The SEM analysis provided conclusive evidence for the presence of a microbial community on the surface of these cultural relics, and subsequent high-throughput sequencing further identified the species composition of the samples. In terms of community structure, Ascomycota is the absolutely dominant group among eukaryotic microorganisms, which is similar to findings from many studies on microbial communities in stone relics [33,34]. Meanwhile, at the phylum level, the next most abundant species is unidentified_Chloroplastida. Ascomycota organisms generally have strong environmental adaptability and the ability to degrade organic matter—characteristics that position them as primary pioneer colonizers on lithic substrates. Lecanoromycetes (Ascomycota) comprises approximately 90% of known lichen fungi, which is the main fungal group in four samples at the class level. The dominant fungal taxa in the remaining three samples are Eurotiomycetes. At the order level, Lecanorales belongs to Lecanoromycetes, and Verrucariales belongs to Eurotiomycetes. Both of these are common lichen fungal groups. The proportions of Verrucariaceae and Ramalinaceae at the family level are almost equal to those of Verrucariales and Lecanorales at the order level. Thus, combined with the sampling photos, the microorganisms on the surface of the relics are shell-like and dense, so it can be concluded that these microorganisms colonizing the surface of the bricks and stones, which have definite macroscopic morphology, are lichens. The high abundance of algae in the sample also supports this, as lichens are complex symbiotic organisms consisting of fungi, the mycobiont, and photosynthetic organisms, the photobiont [35]. It is worth noting that relatively abundant Rotifera and Arthropoda were detected in some samples, among which Rotifera holds significant importance in ecosystem studies. However, current research on these metazoans on lithic artifacts remains relatively scarce.

Actinobacteria (currently Actinomycetota), Proteobacteria (currently Pseudomonadota) and Chloroflexi (currently Chloroflexota) are widely distributed in the prokaryotic community, and the dominance of these four phyla has also been reported in many studies on microbial degradation of stone relics [36,37,38].

Our results have certain limitations. The 18S region in high-throughput sequencing shows high conservation and cannot achieve effective differentiation even at the genus level. Therefore, if lichens in microbial communities are the core research subjects, the ITS region may be a better choice. Subsequent plans include utilizing Funguild for fungal function prediction, combined with microscopic techniques to analyze the extent of stone deterioration caused by microorganisms. During sample collection, attention should be paid to the acquisition of environmental information, which can be used for correlation analysis of environmental factors, thereby enhancing the interpretation of community structural differences and laying a more solid foundation for cultural heritage conservation.

### 4.2. Potential Microorganisms That May Affect Historical Sites

Rock surfaces are typically nutrient-deficient, experience drastic fluctuations in temperature or humidity, have high pH levels, and are frequently exposed to solar radiation, presenting relatively harsh growth substrates and survival conditions for organisms [39,40]. However, fungi, due to their strong enzymatic activity and ability to survive under various extreme conditions such as high salt (5M NaCl), extreme cold (−8 °C), extreme acid (pH 0.6), high radiation (117 kGy), low water activity (aw < 0.582), and oligotrophic conditions [41], can erode various cultural relic materials. Among the cultivable fungi in this study, *Cladosporium*, *Alternaria*, and *Penicillium* have been reported in many studies on stone cultural relics [34,42,43]. Among them, *Cladosporium* can solubilize various minerals through the excretion of gluconic and oxalic acids, posing a severe risk of chromatic alteration [44]. *Aspergillus*, *Penicillium* and other filamentous fungi can produce organic acid that corrodes rocks by dissolving minerals [45,46]. In addition to chemical erosion, physical damage also exists. Many studies have confirmed that after the invasion of filamentous fungal hyphae into the stone matrix, the biological mechanical force formed by their own growth can increase the pressure inside the stone pores, leading to surface crust and peeling [47,48]. Lichens are the main microbial group in the samples we collected, and their effects on stone cultural relics are considered to have both positive and negative aspects. In a study on microbial colonization patterns in Angkor temples, lichens of the genera *Pyxine*, *Lepraria*, and *Cryptothecia* were assessed as having degradation capacity and may potentially reduce water evaporation from stone surfaces through coverage [49]. In another study on biofilms on ceramic artwork surfaces, lichen *Lecanora campestris* and *Protoparmeliopsis muralis* showed shallow penetration without reducing ceramic surface hardness. However, *Verrucaria nigrescens* showed deep penetration, leading to ceramic matrix disintegration and a significant reduction in surface hardness [50].

In addition to lichens, other genera with relatively significant proportions at the genus level of 18S include *Knufia*, *Elliptochloris* and *Trebouxia*. Among them, *Knufia* species are commonly found in severely degraded stone materials, belonging to euendolithic fungi. They can secrete corrosive extracellular substances and have been reported in many studies on the deterioration of stone artifacts [51,52,53]. *Elliptochloris* and *Trebouxia* belong to algae and are commonly found in the green biofilms of stone structures [50,54,55]. In the results of 16S, *Rubrobacter*, *Pseudonocardia*, *Lechevalieria* and *Sphingomonas* are genera with relatively prominent proportions. Among them, *Rubrobacter* and *Lechevalieria* are desiccation-tolerant taxa, which have been identified in a study as bio-indicators of salt efflorescence and desiccation stress in arid environments [56]. In the microbe outbreak on ancient wall paintings in the Maijishan Grottoes, *Rubrobacter* and *Pseudonocardia* were important bacterial communities on mural surfaces [57]. In a study, *Sphingomonas* species were found to produce a yellow pigment to protect themselves from UV rays, which facilitates microbial survival in harsh environments [42].

In this study, only culturable fungi were reported, as our primary focus during sample collection was on eukaryotic organisms such as fungi and algae, resulting in the absence of culturable bacteria. Subsequent studies should include the isolation and purification of culturable bacteria, along with physiological and biochemical characterization of culturable microorganisms to evaluate their potential impact on cultural relics.

### 4.3. Alpha Diversity and Beta Diversity

Samples C1 to C5 were collected from the Qilin Screen Wall, while samples C6 and C7 were obtained from the Text Brick Wall. Specifically, samples C1, C3, and C6 were taken from vertical surfaces, whereas samples C2, C4, C5, and C7 were collected from horizontal surfaces. Based on the alpha diversity results, samples C2, C3, and C4 show high similarity in species richness, evenness, and overall diversity, significantly outperforming the other samples. The species evenness and overall diversity of sample C5 show contrasting patterns between eukaryotes and prokaryotes. Therefore, the location of the samples—whether horizontal or vertical—is not directly correlated with their alpha diversity.

For the beta diversity results, samples C2, C4 and C7 obtained from the horizontal plane showed similarity across both eukaryotic and prokaryotic taxa, with samples C2 and C4 both sourced from the Qilin Screen Wall. Notably, samples C2, C4 and C7 displayed certain morphological similarity. Sample C6 is relatively isolated in both eukaryotic and prokaryotic taxa, and its morphology is indeed the most distinctive. As described in Section 4.1, these microorganisms with specific morphological structures are lichens. Except for sample C6, which appears spherical, the others exhibit flat or shell-like forms. Therefore, it could be hypothesized that on the brick substrate, within the microbial community centered around lichens, the taxa of fungi and bacteria other than lichens exhibit correlations with the species of lichens. Regarding the correlation between location information and beta diversity, a definitive conclusion cannot be drawn due to the limited sample size. In subsequent research, it is necessary to determine whether there are differences in the brick composition between the Qilin Screen Wall and Text Brick Wall, and to investigate the impact of the texture of the qilin motif on microbial colonization.

### 4.4. The Efficacy of Antimicrobials

Given that microorganisms often cause esthetic issues and affect the durability of stone artifacts, it is generally recommended to remove them. Efficient and low-cost broad-spectrum chemical bactericides, including isothiazolinones, quaternary ammonium compounds, sodium hypochlorite, and chlorinated phenolic compounds, have been widely utilized in cultural relics conservation [58,59]. Their disadvantage is that it has a high cost after reusability and is prone to environmental pollution [60]. Plant-derived essential oils and other natural compounds have gained widespread popularity in recent years due to their environmentally friendly properties [61]. In this study, 0.6% antimicrobial BC01 showed promising in vitro antimicrobial activity against four out of six fungi, and the effect was not significantly improved when the concentration was increased to 1.0%. For 1.0% K100, there were three fungi that could be inhibited, while for carvacrol, there were five, and thymol covered all of them. From another perspective, *Cladosporium colombiae* and *Alternaria tenuissima* can be effectively inhibited by almost all antimicrobials. In terms of the antimicrobial effect of nanomaterials, the dispersion of nano-TiO_2_ is better than that of nano-ZnO, but the former is much larger in particle size. The observed differences in antimicrobial efficacy between the two materials may stem from two key factors: First, particle size—smaller particles exhibit superior antimicrobial performance but are prone to aggregation [62]. Second, their distinct mechanisms of action: Nano-TiO_2_ possesses photocatalytic activity that requires UV irradiation, yet the absence of water contact during exposure prevents the generation of free radicals or reactive species. In contrast, nano-ZnO not only facilitates photocatalysis but also releases Zn^2+^ ions for bactericidal effects, while simultaneously inducing oxidative stress through direct interaction with biofilms. While preliminary experiments have confirmed the antimicrobial efficacy of nano-ZnO, numerous studies indicate that the impact of metal ions on stone water absorption remains difficult to determine, which is related to the inherent properties of the stone itself [63]. Regarding the effect on stone surface morphology, research shows that materials containing nano-ZnO maintain color difference below the limit value even after six months of application [64]. Therefore, we intend to subsequently use BC01, carvacrol, and thymol for the next phase of research. Several critical aspects require further investigation. Firstly, considering that the Qilin Screen Wall and Text Brick Wall have undergone a considerable degree of biological weathering, it is imperative to reinforce them. The combination of antimicrobials and reinforcement materials may cause changes in the physical properties and color of the stone, potentially compromising the structural integrity of the cultural relics. Secondly, the impact of these antimicrobials on the esthetic and historical value of the screen wall needs to be carefully evaluated. Any treatment should preserve the unique characteristics and historical significance of these cultural relics without causing unintended damage. Furthermore, the optimal application methods for ensuring uniform distribution and sustained protection of these antimicrobials have not been explored in this study. In summary, it is crucial to determine the long-term efficacy and safety of these antimicrobials in actual cultural relics conservation scenarios.

## 5. Conclusions

This study investigated the Qilin Screen Wall and Text Brick Wall at the Jinshanling Great Wall in Chengde, Hebei, identifying various microorganisms including lichens, molds, and bacteria. Twelve fungal species were isolated. Antimicrobial experiments showed that 0.6% isothiazolinone compound BC01, as well as 50 mg/mL carvacrol and thymol, exhibited certain degrees of antimicrobial efficacy. This research provides a basis for masonry ancient architecture preservation against microbial degradation.

## Figures and Tables

**Table 1:** Grouping and sample correspondence table.

Group	Sample Images	Sample	Sample Location
The Qilin Screen Wall	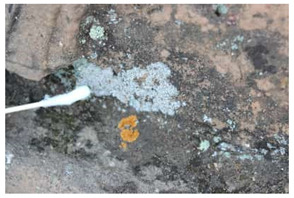	C1	In the lower right corner of the Qilin Screen Wall, on the second brick from the right in the third row. The sample was on a vertical surface.
	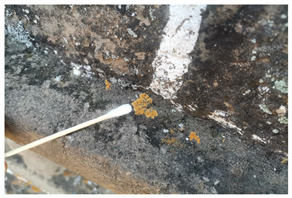	C2	On the lower eave of the Qilin Screen Wall, located at the junction of the second and third bricks from the right in the third row, 25.4 cm to the left of sample C1. The sample was on a horizontal surface.
	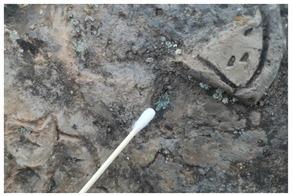	C3	The central–right area of the Qilin Screen Wall, on the first brick from the right in the second row, 60.7 cm above the right of sample C1. The sample was on a vertical surface.
	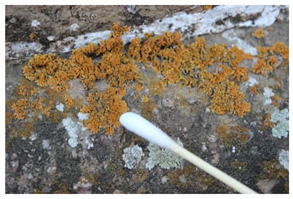	C4	Located at the lower edge of the Qilin Screen Wall base, 54.6 cm below the left of sample C2. The sample was on a horizontal surface.
	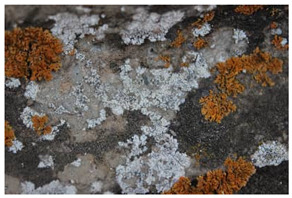	C5	Located on the lower layer of strip bricks of the Qilin Screen Wall base, 19.4 cm above the left of sample C4. The sample was on a horizontal surface.
The Text Brick Wall	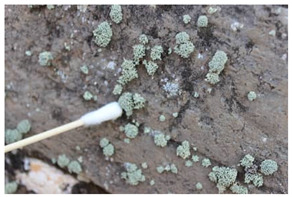	C6	Located on a vertical surface of the Text Brick Wall, 78 cm from the wall base.
	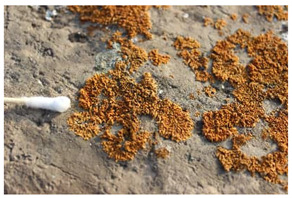	C7	On the top surface of the Text Brick Wall, 36.7 cm above sample C6. The sample was on a horizontal surface.

**Table 2:** The name and concentration of chemical and traditional Chinese medicine antimicrobials (the concentrations of K100 and BC01 are expressed as weight/volume ratios).

Antimicrobial	Concentration		Solvent	Manufacturer
K100	0.6%	1.0%	H_2_O	Beijing Baicheng Wangda Biotechnology Co., Ltd., Beijing, China
BC01	0.6%	1.0%		
Nano-ZnO	50 mg/mL			
Nano-TiO_2_	50 mg/mL			Macklin Biochemical Technology Co., Ltd., Shanghai, China
Carvacrol	50 mg/mL		DMSO	
Geraniol	50 mg/mL			
Thymol	50 mg/mL			

**Table 3:** Identification results of cultivable fungi isolated and purified from the surface of cultural relics.

Strain Number	Microbial Species	Accession Number	Fungal-Derived Samples
JSL-G4	*Aspergillus japonicus*	PX600449	C5
JSL-G5	*Trichoderma harzianum*	PX600450	C5
JSL-G6	*Aspergillus parasiticus*	PX600451	C6
JSL-G7	*Purpureocillium lilacinum*	PX600452	C7
JSL-G9	*Cladosporium colombiae*	PX600453	C2
JSL-G10	*Alternaria tenuissima*	PX600454	C4
JSL-G12	*Penicillium oxalicum*	PX600455	C1
JSL-G13	*Letendraea* sp.	PX600456	C7
JSL-G14	*Nothophoma spiraeae*	PX600457	C2
JSL-G16	*Talaromyces funiculosus*	PX600458	C4
JSL-G17	*Curvularia kusanoi*	PX600459	C6
JSL-G19	*Cephaliophora* sp.	PX600460	C3

**Table 4:** Alpha diversity index statistics of 18S. (chao1: Estimates the total number of species contained in community samples. The higher the number of low-abundance species in the community, the larger the index; dominance: The probability of randomly selecting two sequences from the same sample. The better the evenness of community species, the smaller the index; goods_coverage: Coverage. The higher the sequencing coverage, the larger the index value; observed_features: The number of species observed visually. A higher index indicates more observed species; pielou_e: Evenness index. The higher the evenness of species, the larger the index; Shannon: The total number of taxa and their proportions in the sample. Higher community diversity correlates with a more even species distribution and a larger index. Simpson: A measure of species diversity and evenness within a community. The higher the species evenness, the larger the index.).

Sample_Name	Chao1	Dominance	Goods_Coverage	Observed_Features	Pielou_e	Shannon	Simpson
C1	66.000	0.583	1.000	66	0.265	1.602	0.417
C2	123.200	0.117	1.000	123	0.571	3.965	0.883
C3	124.000	0.267	1.000	124	0.476	3.310	0.733
C4	123.000	0.189	1.000	123	0.496	3.445	0.811
C5	57.000	0.463	1.000	57	0.296	1.726	0.537
C6	76.000	0.490	1.000	76	0.236	1.476	0.510
C7	80.000	0.285	1.000	80	0.401	2.536	0.715

**Table 5:** Alpha diversity index statistics of 16S (the annotations for this table are provided in Table 4).

Sample_Name	Chao1	Dominance	Goods_Coverage	Observed_Features	Pielou_e	Shannon	Simpson
C1	262.207	0.077	0.999	244	0.628	4.984	0.923
C2	834.780	0.012	0.997	808	0.792	7.654	0.988
C3	583.631	0.016	0.998	567	0.777	7.110	0.984
C4	624.655	0.022	0.997	600	0.746	6.883	0.978
C5	347.588	0.022	1.000	347	0.779	6.570	0.978
C6	216.000	0.071	1.000	216	0.630	4.885	0.929
C7	374.650	0.063	0.998	351	0.661	5.586	0.937

## Data Availability

The datasets presented in this study can be found in online repositories. The names of the repository/repositories and accession number(s) can be found in the article.

## Supplementary Materials

The following supporting information can be downloaded at https://www.mdpi.com/article/10.3390/microorganisms14051056/s1, Figure S1: Rarefaction curves of 18S; Figure S2: Rarefaction curves of 16S; Figure S3: The network of prokaryotic association analysis; Table S1: The percentage of the top ten eukaryotic taxa by relative abundance at each taxonomic level; Table S2: The percentage of the top ten prokaryotic taxa by relative abundance at each taxonomic level.

## References

[1] Yang Y., Zhang Y., Li Y. (2024). Temporal and spatial distribution characteristics of the Ming Great Wall. Herit. Sci..

[2] Dettmering T., Dai S. (2022). Types of lime binders in mortars used for the construction of the Ming Great Wall of China and their importance for the development of a conservation strategy. Built Herit..

[3] Sanmartín P., DeAraujo A., Vasanthakumar A. (2018). Melding the old with the new, Trends in methods used to identify, monitor, and control microorganisms on cultural heritage materials. Microb. Ecol..

[4] Geweely N.S. (2023). New frontiers review of some recent conservation techniques of organic and inorganic archaeological artefacts against microbial deterioration. Front. Microbiol..

[5] Warscheid T., Braams J. (2000). Biodeterioration of stone, A review. Int. Biodeterior. Biodegrad..

[6] Lisci M., Monte M., Pacini E. (2003). Lichens and higher plants on stone, A review. Int. Biodeterior. Biodegrad..

[7] Ding X., Lan W., Yan A., Li Y., Katayama Y., Gu J. (2021). Microbiome characteristics and the key biochemical reactions identified on stone world cultural heritage under different climate conditions. J. Environ. Manag..

[8] Miller A.Z., Sanmartín P., Pereira-Pardo L., Dionísio A., Saiz-Jimenez C., Macedo M.F., Prieto B. (2012). Bioreceptivity of building stones, A review. Sci. Total Environ..

[9] Li Y., Gu J. (2022). A more accurate definition of water characteristics in stone materials for an improved understanding and effective protection of cultural heritage from biodeterioration. Int. Biodeterior. Biodegrad..

[10] Vázquez-Nion D., Silva B., Prieto B. (2018). Influence of the properties of granitic rocks on their bioreceptivity to subaerial phototrophic biofilms. Sci. Total Environ..

[11] Liu X., Koestler R.J., Warscheid T., Katayaman Y., Gu J. (2020). Microbial deterioration and sustainable conservation of stone monuments and buildings. Nat. Sustain..

[12] Chen X., Bai F., Huang J., Lu Y., Wu Y., Yu J., Bai S. (2021). The Organisms on Rock Cultural Heritages, Growth and Weathering. Geoheritage.

[13] Jang K., Viles H. (2022). Moisture Interactions Between Mosses and Their Underlying Stone Substrates. Stud. Conserv..

[14] Pinna D. (2014). Biofilms and lichens on stone monuments, do they damage or protect?. Front. Microbiol..

[15] Zhao J., Csetenyi L., Gadd G.M. (2021). Fungal-induced CaCO_3_ and SrCO_3_ precipitation, A potential strategy for bioprotection of concrete. Sci. Total Environ..

[16] Cao Y., Bowker M.A., Delgado-Baquerizo M., Xiao B. (2023). Biocrusts protect the Great Wall of China from erosion. Sci. Adv..

[17] Wang T., Guo Q., Pei Q., Chen W., Wang Y., Zhang B., Yu J. (2023). Destruction or protection? Experimental studies on the mechanism of biological soil crusts on the surfaces of earthen sites. Catena.

[18] Liu X., Qian Y., Wu F., Wang Y., Wang W., Gu J. (2022). Biofilms on stone monuments, biodeterioration or bioprotection?. Trends Microbiol..

[19] Camara B., De los Rios A., Urizal M., Alvarez de Buergo M., Jose Varas M., Fort R., Ascaso C. (2011). Characterizing the microbial colonization of a dolostone quarry implications for stone biodeterioration and response to biocide treatments. Microb. Ecol..

[20] Fidanza M.R., Caneva G. (2019). Natural biocides for the conservation of stone cultural heritage, A review. J. Cult. Herit..

[21] Zhu C., Wang L., Wang B., Wang B., Tang M., Wang X., Li Q., Hu Y., Zhang B. (2023). Application and evaluation of a new blend of biocides for biological control on cultural heritages. Int. Biodeterior. Biodegrad..

[22] Wang Y., Wang C., Hou L., Yang X., Li C., Cui S., Ma C., Wang L., Zhang L., Liu Y. (2024). Microbial Diversity and Biodegradation Mechanism of Microorganisms in the Dingtao M2 Tomb. Int. J. Mol. Sci..

[23] Caporaso J.G., Lauber C.L., Walters W.A., Berg-Lyons D., Lozupone C.A., Turnbaugh P.J., Fierer N., Knight R. (2011). Global patterns of 16S rRNA diversity at a depth of millions of sequences per sample. Proc. Natl. Acad. Sci. USA.

[24] Cheung M.K., Au C.H., Chu K.H., Kwan H.S., Wong C.K. (2010). Composition and genetic diversity of picoeukaryotes in subtropical coastal waters as revealed by 454 pyrosequencing. ISME J..

[25] Price M.N., Dehal P.S., Arkin A.A. (2009). FastTree, computing large minimum evolution trees with profiles instead of a distance matrix. Mol. Biol. Evol..

[26] Lozupone C., Knight R. (2005). UniFrac, a new phylogenetic method for comparing microbial communities. Appl. Environ. Microbiol..

[27] Lozupone C., Lladser M.E., Knights D., Stombaugh J., Knight R. (2011). UniFrac, an effective distance metric for microbial community comparison. ISME J..

[28] Lozupone C.A., Hamady M., Kelley S.T., Knight R. (2007). Quantitative and qualitative beta diversity measures lead to different insights into factors that structure microbial communities. Appl. Environ. Microbiol..

[29] White T.J., Bruns T., Lee S., Taylor J.W., Innis M.A., Gelfand D.H., Sninsky J.J., White T.J. (1990). Amplification and direct sequencing of fungal ribosomal RNA genes for phylogenetics. PCR Protocols, a Guide to Methods and Applications.

[30] Wang Y., Wang Y., Guan Z., Wang Z., Duan Y., Min C., Zhong Y., Hou L., Pan J. (2025). Analysis and prevention of microbial degradation of shadow puppetry artifacts preserved in the National Shadow Puppetry Museum in Chengdu. Front. Microbiol..

[31] Li B., Zhang X., Guo F., Wu W., Zhang T. (2013). Characterization of tetracycline resistant bacterial community in saline activated sludge using batch stress incubation with high-throughput sequencing analysis. Water Res..

[32] Wang Y., Duan Y., Wang Y., Wang C., Hou L., Min C., Zhong Y., Shi G., Pan J. (2025). Microbial analysis in the preservation environments of shadow puppet cultural relics in museum. npj Herit. Sci..

[33] Zhang Y., Wu F., Su M., He D., Gu J.-D., Guo Q., Kakakhel M.A., Yang Y., Wang W., Feng H. (2021). Spatial and temporal distributions of microbial diversity under natural conditions on the sandstone stelae of the Beishiku Temple in China. Int. Biodeterior. Biodegrad..

[34] Qiao S., Wang Z., Zhang R., Wang Y., Wang C., Gao G., Pan J. (2025). Microbial Community Analysis and Environmental Association in Cave 6 of the Yungang Grottoes. Microorganisms.

[35] Cozzolino A., Adamo P., Bonanomi G., Motti R. (2022). The Role of Lichens, Mosses, and Vascular Plants in the Biodeterioration of Historic Buildings, A Review. Plants.

[36] Wu F. (2024). Study of Microbiomes and Bioweathering Mechanisms on Sandstone Cultural Heritage of Beishiku Temple in China. Lanzhou University.

[37] Li Q., Zhang B., Yang X., Ge Q. (2018). Deterioration-Associated Microbiome of Stone Monuments, Structure, Variation, and Assembly. Appl. Environ. Microbiol..

[38] He J., Zhang N., Muhammad A., Shen X., Sun C., Li Q., Hu Y., Shao Y. (2022). From surviving to thriving, the assembly processes of microbial communities in stone biodeterioration, A case study of the West Lake UNESCO World Heritage area in China. Sci. Total Environ..

[39] Louati M., Ennis N.J., Ghodhbane-Gtari F., Hezbri K., Sevigny J.L., Fahnestock M.F., Cherif-Silini H., Bryce J.G., Tisa L.S., Gtari M. (2019). Elucidating the ecological networks in stone-dwelling microbiomes. Environ. Microbiol..

[40] Zanardini E., May E., Purdy K.J., Murrell J.C. (2019). Nutrient cycling potential within microbial communities on culturally important stoneworks. Environ. Microbiol. Rep..

[41] Coleine C., Stajich J.E., Selbmann L. (2022). Fungi are key players in extreme ecosystems. Trends Ecol. Evol..

[42] Zhang Y., Su M., Wu F., Gu G., Li J., He D., Guo Q., Cui H., Zhang Q., Feng H. (2023). Diversity and Composition of Culturable Microorganisms and Their Biodeterioration Potentials in the Sandstone of Beishiku Temple, China. Microorganisms.

[43] Wu F., Li J. (2022). The Current Status and Future Prospects for Microbial Research on Stone Cultural Relics. Res. Conserv. Cave Temples Earthen Sites.

[44] Pyzik A., Ciuchcinski K., Dziurzynski M., Dziewit L. (2021). The bad and the good—Microorganisms in cultural heritage environments—An update on biodeterioration and biotreatment approaches. Materials.

[45] Salvadori O., Municchia A.C. (2016). The Role of Fungi and Lichens in the Biodeterioration of Stone Monuments. Open Conf. Proc. J..

[46] Ma W., Wu F., Tian T., He D., Zhang Q., Gu J., Duan Y., Ma D., Wang W., Feng H. (2020). Fungal diversity and its contribution to the biodeterioration of mural paintings in two 1700-year-old tombs of China. Int. Biodeterior. Biodegrad..

[47] Isola D., Zucconi L., Onofri S., Caneva G., Hoog G.S., Selbmann L. (2016). Extremotolerant rock inhabiting black fungi from Italian monumental sites. Fungal Divers..

[48] Moroni B., Pitzurra L. (2008). Biodegradation of atmospheric pollutants by fungi, A crucial point in the corrosion of carbonate building stone. Int. Biodeterior. Biodegrad..

[49] Bartoli F., Municchia A.C., Futagami Y., Kashiwadani H., Moon K.H., Caneva C. (2014). Biological colonization patterns on the ruins of Angkor temples (Cambodia) in the biodeterioration vs bioprotection debate. Int. Biodeterior. Biodegrad..

[50] Pinna D., Mazzotti V., Gualtieri S., Voyron S., Andreotti A., Favero-Longo S.E. (2023). Damaging and protective interactions of lichens and biofilms on ceramic *dolia* and sculptures of the International Museum of Ceramics, Faenza, Italy. Sci. Total Environ..

[51] Breitenbach R., Silbernagl D., Toepel J., Sturm H., Broughton W.J., Sassaki G.L., Gorbushina A.A. (2018). Corrosive extracellular polysaccharides of the rock-inhabiting model fungus *Knufia petricola*. Extremophiles.

[52] Leo F.D., Antonelli F., Pietrini A.M., Ricci S., Urzì C. (2019). Study of the euendolithic activity of black meristematic fungi isolated from a marble statue in the Quirinale Palace’s Gardens in Rome, Italy. Facies.

[53] Rizk S.M., Magdy M., Leo F.D., Werner O., Rashed M.A., Ros R.M., Urzì C. (2023). Culturable and unculturable potential heterotrophic microbiological threats to the oldest pyramids of the Memphis necropolis, Egypt. Front. Microbiol..

[54] Cutler N.A., Oliver A.E., Viles H.A., Ahmad S., Whiteley A.S. (2013). The characterisation of eukaryotic microbial communities on sandstone buildings in Belfast, UK, using TRFLP and 454 pyrosequencing. Int. Biodeterior. Biodegrad..

[55] Rifón-Lastra A., Noguerol-Seoane Á. (2001). Green algae associated with the granite walls of monuments in Galicia (NW Spain). Cryptogam. Algol..

[56] Mohammadipanah F., Wink J. (2016). Actinobacteria from arid and desert habitats, Diversity and biological activity. Front. Microbiol..

[57] He D., Wu F., Ma W., Zhang Y., Gu J., Duan Y., Xu R., Feng H., Wang W., Li S. (2021). Insights into the bacterial and fungal communities and microbiome that causes a microbe outbreak on ancient wall paintings in the Maijishan Grottoes. Int. Biodeterior. Biodegrad..

[58] Kakakhel M.A., Wu F., Gu J., Feng H., Shah K., Wang W. (2019). Controlling biodeterioration of cultural heritage objects with biocides, A review. Int. Biodeterior. Biodegrad..

[59] Romani M., Warscheid T., Nicole L., Marcon L., Martino P.D., Suzuki M.T., Lebaron P., Lami R. (2022). Current and future chemical treatments to fight biodeterioration of outdoor building materials and associated biofilms, Moving away from ecotoxic and towards efficient, sustainable solutions. Sci. Total Environ..

[60] Scheerer S., Ortega-Morales O., Gaylarde C. (2009). Microbial Deterioration of Stone Monuments—An Updated Overview. Adv. Appl. Microbiol..

[61] Li T., Zhang H., Tan X., Zhang R., Wu F., Yu Z., Su B. (2024). Antimicrobial Activities of Medicinal Plants and Their Application for Controlling Microbial Deterioration at Cultural Heritage Sites. Res. Conserv. Cave Temples Earthen Sites.

[62] Huang X., Jia M., Zhang M. (2021). Research progress on the application of nanomaterials in the treatment of biological diseases of stone cultural relics. China Cult. Herit..

[63] Munafò P., Goffredo G.B., Quagliarini E. (2015). TiO_2_-based nanocoatings for preserving architectural stone surfaces, An overview. Constr. Build. Mater..

[64] Van der Werf I.D., Ditaranto N., Picca R.A., Sportelli M.C., Sabbatini L. (2015). Development of a novel conservation treatment of stone monuments with bioactive nanocomposites. Herit. Sci..

