# Supplementary material for: Analysis of Microbial Communities and Microbial Preservation of the Qilin Screen Wall and Text Brick Wall in the Jinshanling Great Wall
Source: Microorganisms. 2026 May 8;14(5):1056. doi: 10.3390/microorganisms14051056 (PMC13210244; doi:10.3390/microorganisms14051056)
Supplement: Supplementary file 1 [file microorganisms-14-01056-s001.zip › Supplementary Material.pdf]

Supplementary Material

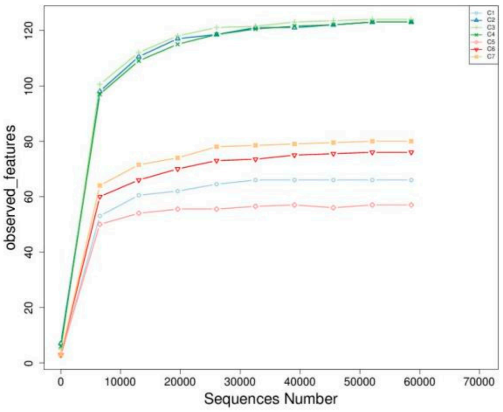

(a)

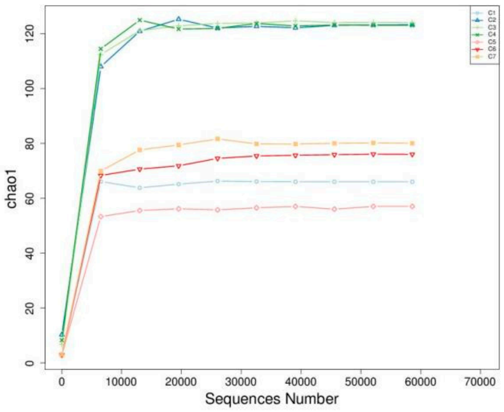

(b)

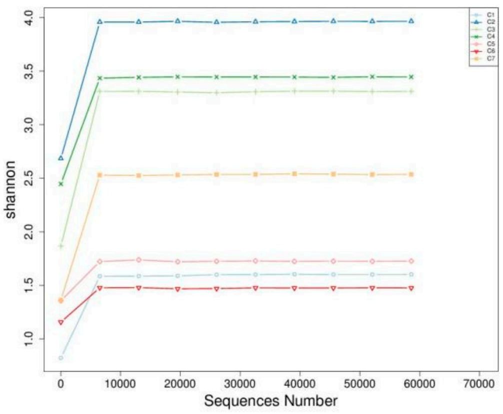

(c)

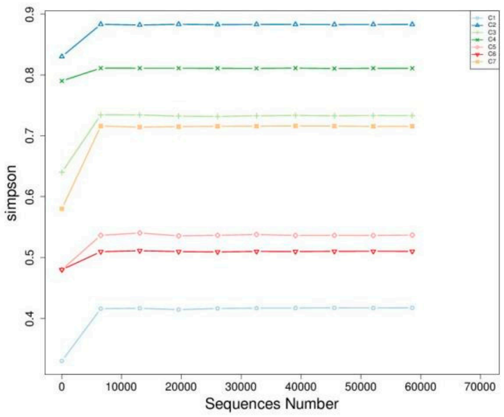

(d)

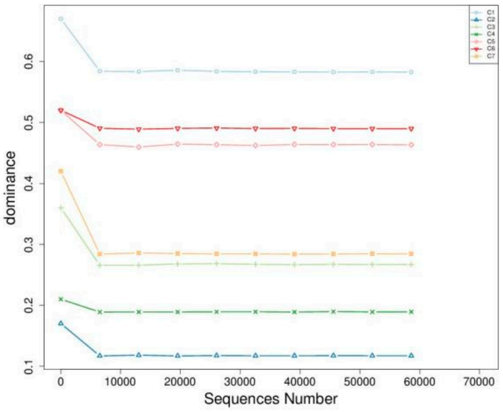

(e)

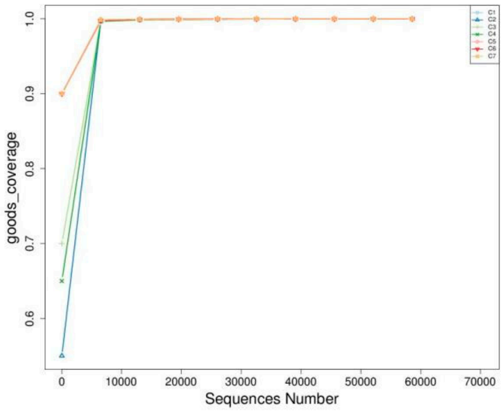

(f)

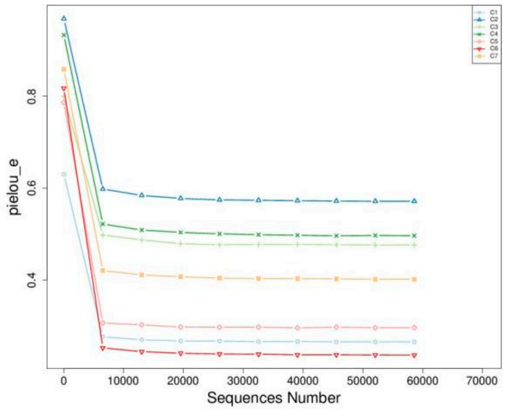

(**g**)

Figure S1. Rarefaction curves of 18S. (a): observed_features; (b): chao1; (c): shannon; (d): simpson; (e): dominance; (f): goods_coverage; (g): pielou_e. (**observed_features**: The number of species observed visually. A higher index indicates more observed species; **chao1**: Estimates the total number of species contained in community samples. The higher the number of low-abundance species in the community, the larger the index; **shannon**: The total number of taxa and their proportions in the sample. Higher community diversity correlates with more even species distribution and a larger index; **simpson**: A measure of species diversity and evenness within a community. The higher the species evenness, the larger the index; **dominance**: The probability of randomly selecting two sequences from the same sample. The better the evenness of community species, the smaller the index; **goods_coverage**: Coverage. The higher the sequencing coverage, the larger the index value; **pielou_e**: Evenness index. The higher the evenness of species, the larger the index.)

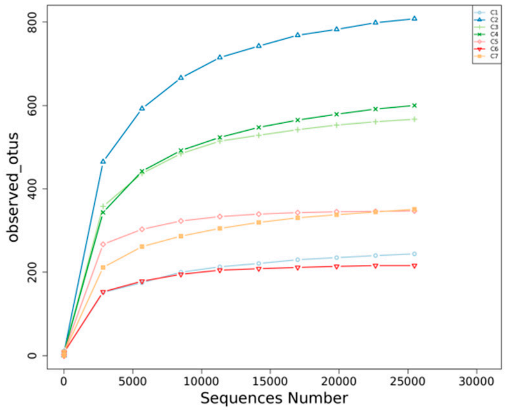

(**a**)

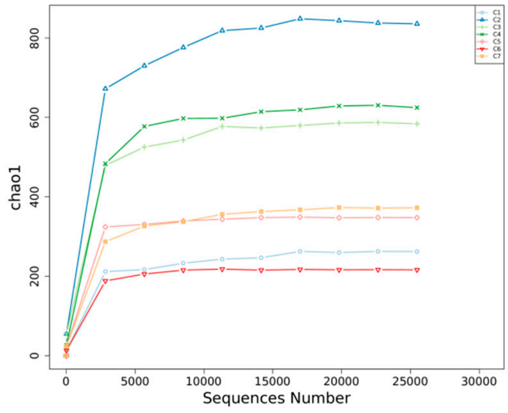

(**b**)

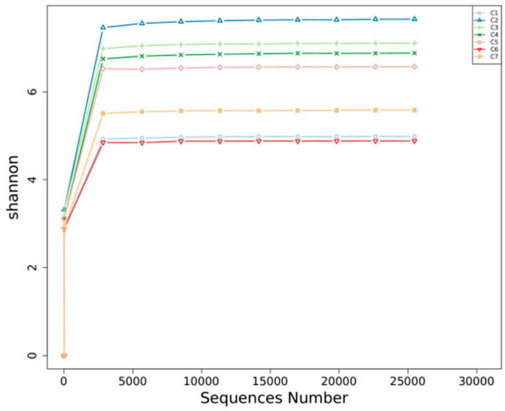

(**c**)

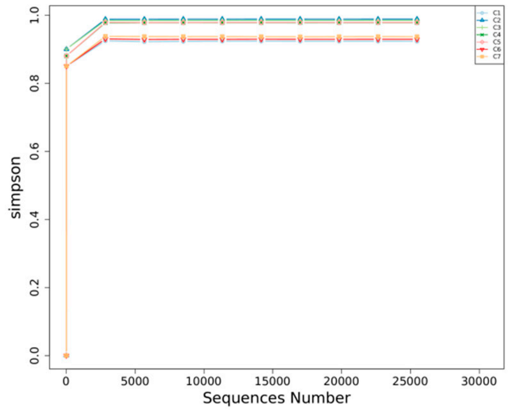

(**d**)

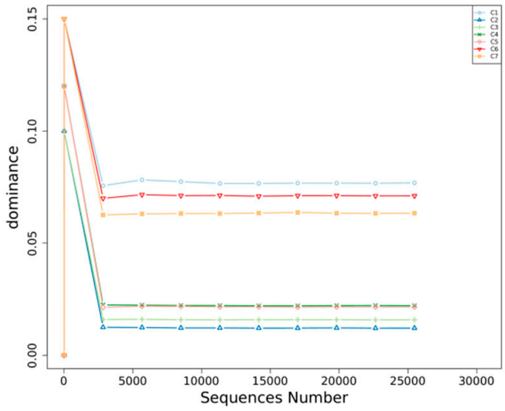

(**e**)

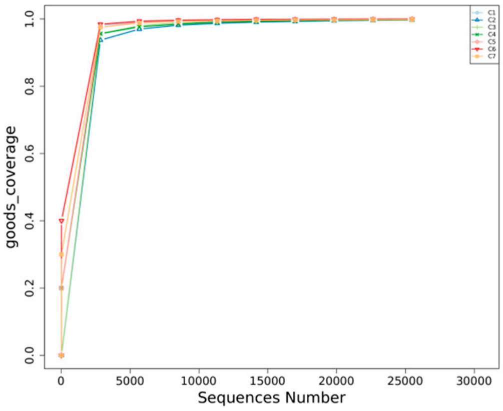

(**f**)

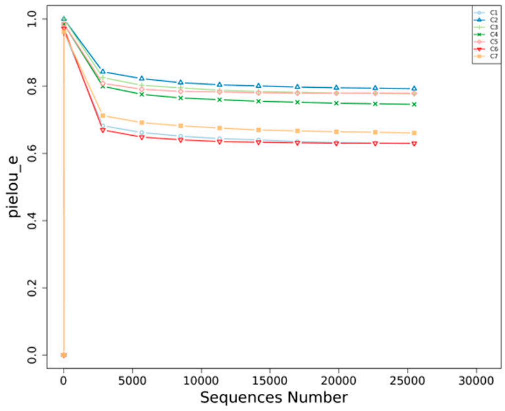

(**g**)

Figure S2. Rarefaction curves of 16S. (a): observed_features; (b): chao1; (c): shannon; (d): simpson; (e): dominance; (f): goods_coverage; (g): pielou_e.

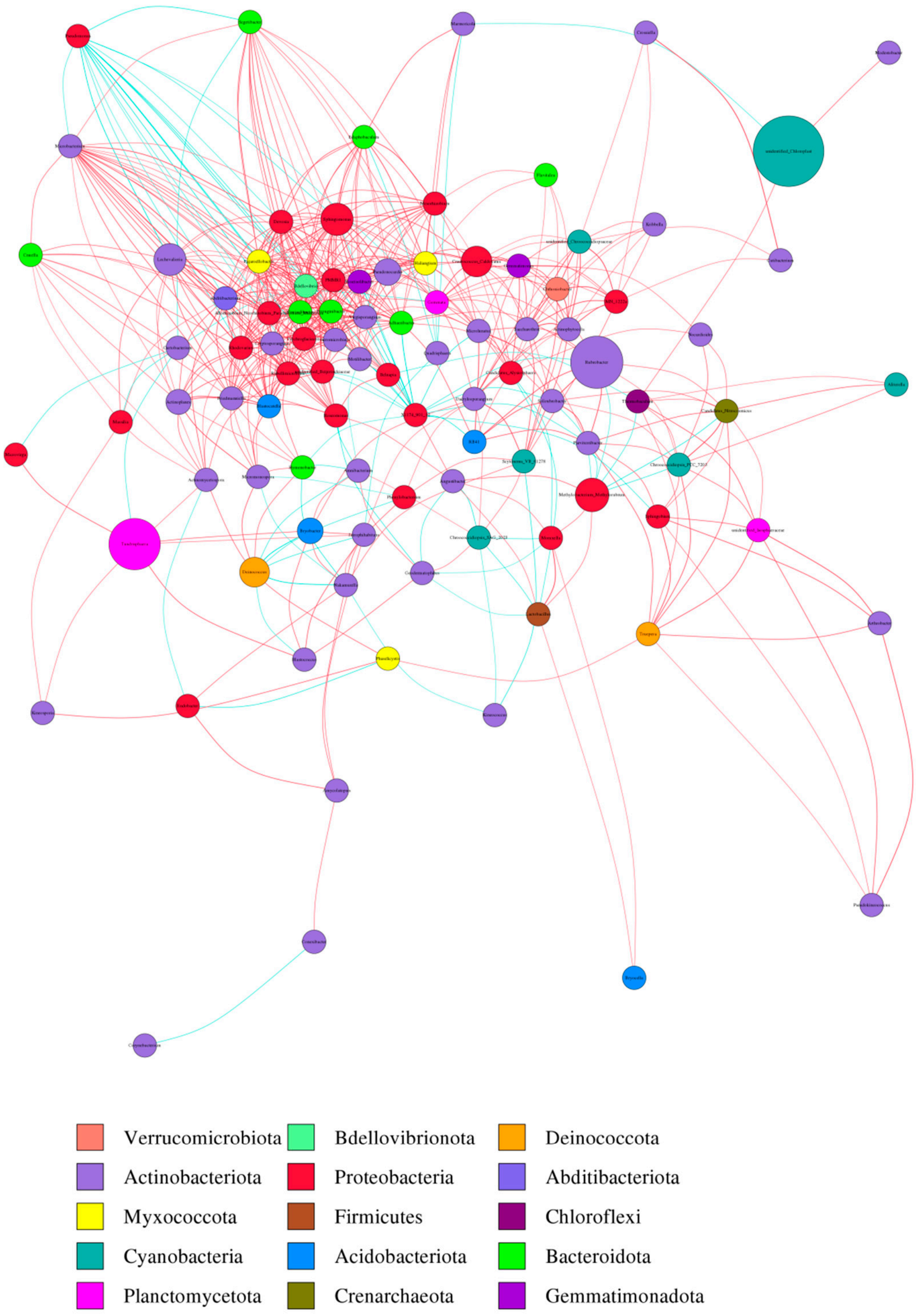

Figure S3. The network of prokaryotic association analysis (Different nodes represent different genera, and node size represents the average relative abundance of that genus. Nodes in the same phylum have the same color (as shown in the legend). The thickness of the connections between nodes is positively correlated with the absolute value of the correlation coefficient between species interactions, and the color of the connections corresponds positively or negatively to the correlation (red positive correlation, blue negative correlation).
